# Role of Targeted Therapy in the Management of von Hippel-Lindau Disease-Associated Renal Cell Carcinoma: A Single-Proportion Meta-Analysis

**DOI:** 10.7759/cureus.97606

**Published:** 2025-11-23

**Authors:** Adeyemi Abimbola, Adewale Ayeni, Ayoola Olaleye, Avanti Banerjee, Ananda Dhanasekaran

**Affiliations:** 1 Clinical Sciences, University of Edinburgh, Edinburgh, GBR; 2 General Surgery, Queen Elizabeth Hospital Birmingham, Birmingham, GBR; 3 Urology, Queen Elizabeth Hospital Birmingham, Birmingham, GBR; 4 Urology, University of Birmingham, Birmingham, GBR

**Keywords:** efficacy, renal cell carcinoma, targeted therapy, toxicity, von hippel-lindau syndrome

## Abstract

Management of renal cell carcinoma (RCC) associated with von Hippel-Lindau (VHL) disease is challenging due to multifocality and the risk of renal failure from repeated surgery. Recent advances in targeted therapy directed at the hypoxia-inducible factor (HIF)-2α and vascular endothelial growth factor receptor (VEGFR) pathways offer new therapeutic options. This meta-analysis evaluated the overall response and toxicity of targeted therapy in VHL-associated RCC.

A systematic literature search was conducted in PubMed, Embase, Cochrane, and Scopus databases from January 2000 to January 2024, following the Preferred Reporting Items for Systematic Reviews and Meta-Analyses (PRISMA) guidelines. Eligible studies included clinical trials and retrospective analyses reporting the efficacy and adverse events of systemic targeted therapies in VHL-related RCC. Pooled response rates and confidence intervals (CI) were calculated using a random-effects model, with heterogeneity assessed by I² statistics.

Five studies (188 RCC cases) met the inclusion criteria. The pooled overall response rate to targeted therapy was 42% (95% CI: 28-56). Subgroup analysis showed higher efficacy of HIF-2α inhibitors (belzutifan) compared to VEGFR inhibitors (62% vs. 44%; 95% CI: 49-74 and 30-59, respectively). Heterogeneity was moderate (I²=68%). Most adverse events were grades 1-2, with better tolerability in HIF-2α inhibitors.

Targeted therapy focusing on the molecular pathogenesis of VHL-associated RCC provides meaningful disease control while preserving renal function. HIF-2α inhibitors demonstrate superior efficacy and lower toxicity compared with VEGFR-targeted agents, representing a promising non-surgical alternative for managing VHL-related RCC.

## Introduction and background

von Hippel-Lindau (VHL) disease is a rare genetic disorder arising as a result of a germline mutation in the gene coding the VHL protein located at the short arm of chromosome 3 (3p25) [1]. It is an autosomal dominant disease with a high penetrance reported to approach 100% by age 60 years [2]. This disorder is characterized by hereditary multi-organ benign and malignant neoplasms, including clear cell renal cell carcinoma (RCC), hemangioblastoma, pancreatic neuroendocrine tumor, pheochromocytoma, paraganglioma, and multiple renal and pancreatic cysts [3].

The incidence of VHL disease is approximately 1:40,000 live births, and the lifetime risk of developing RCC in genetically mutated VHL individuals is approximately 70% [3,4]. In a retrospective study examining the causes of mortality among Danish individuals with VHL disease, hemangioblastoma emerged as the leading cause of death. RCC was the second most frequent contributor, accounting for 36% of VHL-related deaths. Notably, affected patients typically develop their first RCC between the ages of 20 and 40. Characteristically, the biology of these tumors differs from sporadic renal cancer in that they are often solid, bilateral, and multifocal and are often associated with multiple renal cysts with malignant potential [4,5].

Over the years, the gold standard of care has included active surveillance for small lesions and nephron-sparing surgical procedures when the lesion grows beyond 3 cm due to the considerable risk of metastatic disease [4]. The main challenge of repeated renal surgery for VHL-related RCC is progressive nephron loss leading to end-stage renal disease with a significantly increased risk of death, cardiovascular events, and dialysis-related hospitalization [4,6,7]. Patients with VHL-related tumors characteristically develop early metastatic potential when compared with sporadic renal tumors and thus are subjected to repeated surgeries for complete excision [4,5].

In recent times, the challenges of renal loss shifted the aim of treating VHL-related RCC from the complete surgical excision of tumors to the prevention of metastasis and the conservation of renal parenchyma [8]. A literature review on the relationship between tumor size and the metastatic potential of VHL-related RCC found no metastasis in tumors less than 3 cm [9,10]. Duffey et al., in a prospective evaluation of the natural history of 73 patients with tumor size greater than 3 cm, found that 27.4% of their study population developed metastasis [4]. Although an increasing number of studies indicate that surgical intervention may be safely postponed until lesions attain a diameter of 4 cm, the existing evidence base remains constrained by methodological limitations, such as the absence of control groups, small cohort sizes, and potential biases. As a result, these conclusions have yet to gain broad acceptance within the clinical community. To this end, surgical intervention has traditionally been offered to patients with a tumor size >3 cm, which would eventually result in end-stage renal disease and death from treatment-related complications.

Molecular targeted therapy that blocks cancer growth and spread has revolutionized cancer therapeutics since its discovery. The identification and blockage of an ideal target in cancer tumorigenesis are central to the success of cancer treatment [11]. This identification is achievable through cancer genomic sequencing that differentiates normal tissues from abnormal cancer cells [12,13]. Targets, which are therefore selected for targeted therapy, are molecules that are directly or indirectly involved in tumor growth and spread, and these include signaling molecules, cell cycle proteins, growth factors, and molecules that influence apoptosis and promote angiogenesis [14]. The understanding of this molecular basis that governs renal tumorigenesis has progressed with the identification of the VHL gene. This tumor suppressor gene controls the groups of transcriptional proteins responsible for cellular proliferation. Localized on the short arm of chromosome 3 locus 25 (3p25), this gene encodes proteins that direct cellular response to hypoxia by downregulating the family of hypoxic-inducible factor (HIF) transcription factors through which VHL inactivation mediates carcinogenesis [2,3].

HIF exists in two isoforms, that is, HIF-1α and HIF-2α, and has been shown to control multiple aspects of tumorigenesis, including the activation of genes that regulate cellular proliferation, angiogenesis, and cell survival. While HIF-1α is broadly active in a variety of hypoxic tissues, HIF-2α is restricted to the kidney, lung, and heart [15]. Overexpression of HIF-2α is seen in VHL-defective RCC [16]. In normoxic conditions, the VHL protein acts as an E3 ubiquitin ligase, forming a complex with the alpha subunits of HIF, with the subsequent proteolysis of HIF [17]. Under hypoxic conditions or following the inactivation of the VHL gene, the oxygen-dependent alpha subunit of HIF becomes stable and forms a complex HIF transcription factor by binding to the constitutively expressed HIF-β unit. This complex transcriptional factor activates groups of tumor-specific genes, such as angiogenic factors vascular endothelial growth factor A (VEGF-A) and platelet-derived growth factor B (PDGF-B) and growth factors transforming growth factor alpha (TGF-α), glucose transporter 1, erythropoietin, and cyclin D1 [17]. The understanding of the critical role of the VHL protein and the downstream regulation of various genes led to the development of molecular targeted therapy in renal cancer management, and response to this therapy will eventually reduce the burden posed by nephron-sparing surgical procedures through the conservation of sufficient renal function and by improving overall survival without the undue risk of metastasis [7].

Objective

Although the last two decades saw a significant development of several targeted therapies in VHL-related RCC, there exists no high-quality evidence designed to appraise the available targeted therapies. This study aims to resolve this by evaluating the overall response rate of targeted therapy in VHL-associated RCC through a systematic review of available literature.

## Review

Methods

We performed a literature search to identify all published studies, including case series, observational studies, and open-label studies, investigating the role of targeted therapies in the management of VHL-associated RCC. This meta-analysis was conducted using the Preferred Reporting Items for Systematic Reviews and Meta-Analyses (PRISMA) guidelines [18].

Inclusion Criteria

Included in the meta-analysis were (1) studies that included adult participants (>18 years), with germline mutations in the VHL gene and a histological diagnosis of RCC measured with computed tomography (CT) or magnetic resonance imaging (MRI) as defined by the Response Evaluation Criteria in Solid Tumors (RECIST), (2) studies that included patients treated with systemic targeted therapy, and (3) studies written in English and published in peer-reviewed journals Both retrospective and non-controlled open-label trials were included.

Exclusion Criteria

Excluded from the study were (1) case reports or studies evaluating the treatment response in VHL disease patients without measurable response in RCC and (2) studies in which systemic targeted therapy was not used and/or studies where response rates were not reported.

Information Sources and Searches

Two independent reviewers performed a comprehensive electronic search across databases, including PubMed, Embase, Cochrane, and Scopus, from January 2000 to January 2024. The following basic search terms were used, both alone and in combination: "Von Hippel-Lindau associated renal cell carcinoma OR VHL related renal cell carcinoma OR VHL associated renal cell carcinoma OR Von-Hippel Lindau associated renal cancer or VHL associated RCC AND Targeted therapy OR biologic therapy". Reference lists of the retrieved articles and relevant articles were examined for cross-references.

Selection Criteria, Title, and Abstract Screening

Titles and abstracts were independently examined by two of the authors. Eligibility criteria were applied, and a list of full texts was developed through consensus. This selection process refined the number of relevant articles to five (two retrospective and three open-label trials). The article by Oudard et al. was excluded as no clear definition of response was reported.

Data Extraction

All data were extracted independently by the author, and any disagreement was resolved with the supervisor's consultation. Data extracted were entered into an Excel worksheet (Microsoft Corporation, Redmond, Washington, United States). The following data were extracted: author of the article and year of publication, the countries involved in the primary research, journal of publication, title of the study, type of study design, inclusion and exclusion criteria, numbers of RCC treated in each article, duration of treatment with targeted therapy and types of targeted therapy used, duration of follow-up, overall response rate, and average adverse events.

Meta-Analysis

All extracted data from the studies included were entered into RStudio 2024.04.1+748 "Chocolate Cosmos" (R Foundation for Statistical Computing, Vienna, Austria). We defined our primary outcome and the overall response rate of RCC in VHL to targeted therapy according to the included study investigators' operational definitions of response as provided in the original reports. We calculated the pooled proportion of RCC that responded using pre-determined criteria across all included studies, together with 95% CI.

We anticipate heterogeneity due to methodological variability in the included studies; hence, a random-effects meta-analysis was employed. Observed heterogeneity was computed using I^2^ statistics [19]. Publication bias was assessed using the visual inspection of funnel plots and the Egger bias test [20].

Results

Literature Search Results

**Figure 1:**
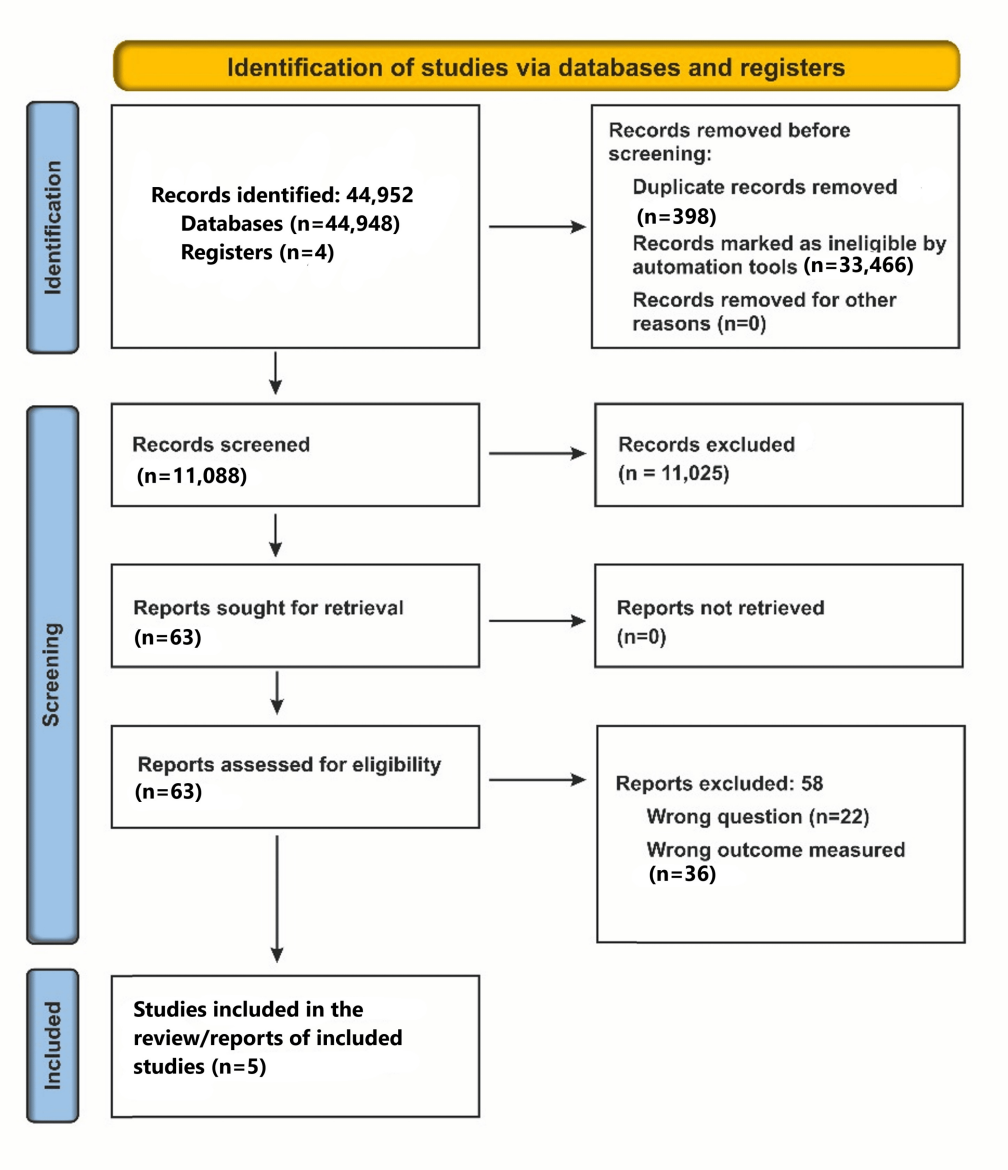
PRISMA flow diagram of article search and review process PRISMA: Preferred Reporting Items for Systematic Reviews and Meta-Analyses

Our initial search yielded 44,952 studies, out of which 44,889 articles were excluded based on relevance and eligibility. A further 58 articles were excluded due to wrong questions or outcome measures. Finally, five articles qualified. Figure 1 depicts the PRISMA flow diagram.

Characteristics of the Included Articles

We included five studies in this systematic review. Included studies were published between 2011 and 2021. The studies were conducted in the United States, the United Kingdom, Denmark, France, Italy, and China. One hundred and eighty-eight RCC were treated with targeted therapy with a median follow-up duration ranging from 11 to 39.4 months. Response rate was assessed using RECIST.

Overall Response Rate

**Figure 2:**
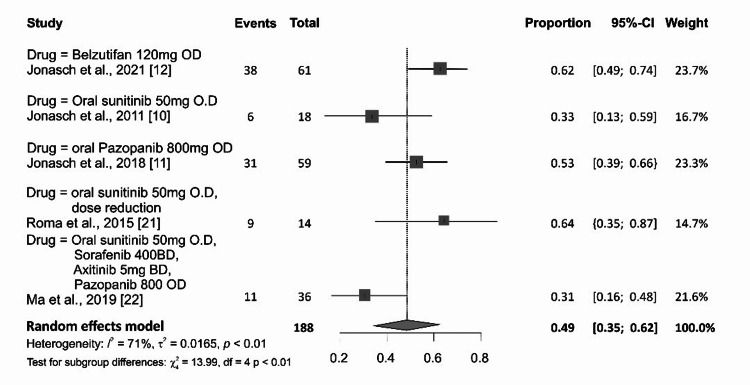
Meta-analysis of the overall response rate of targeted therapy

**Figure 3:**
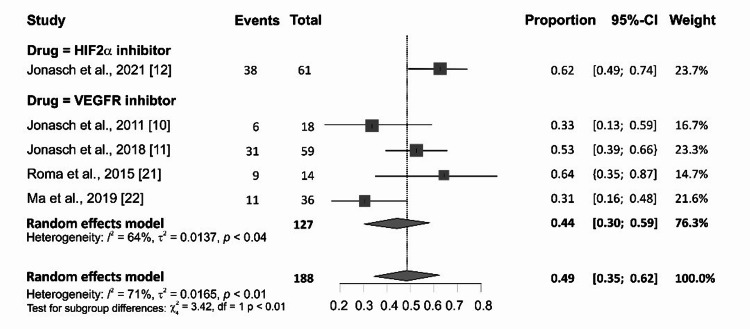
Subgroup analysis of HIF-2a vs. VEGFR HIF: hypoxia-inducible factor; VEGFR: vascular endothelial growth factor receptor

**Figure 4:**
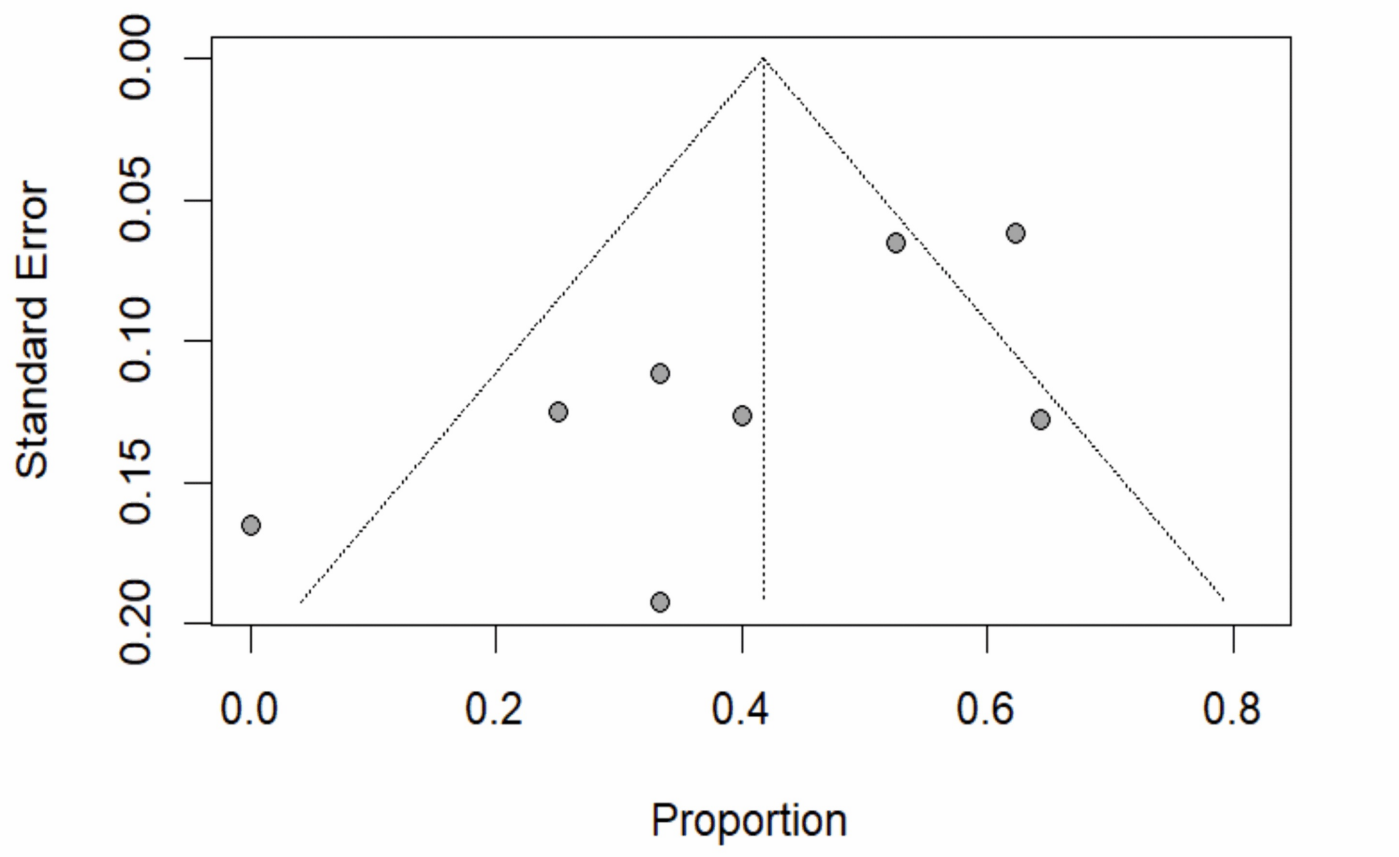
Funnel plot for main analysis

It was possible to pool data on the proportion of the overall response rate across 188 RCC patients who were treated with targeted therapy in the five studies, which include three single-arm open-label trials and two retrospective studies. Overall, the proportion of RCC with response to targeted therapy was 0.42 (95% CI: 0.28-0.56). There was evidence of heterogeneity (p<0.01; I^2^=68%) as shown in Figure 2. Response to belzutifan, an HIF-2α inhibitor, was higher than VEGFR inhibitor at 0.62 (95% CI: 0.49-0.74) and 0.44 (95% CI: 0.30-0.59), respectively, in the subgroup analysis (Figure 3). Risk of publication bias was assessed using the Egger bias test and funnel plot, as shown in Figure 4.

Adverse Drug Event

The severity of adverse events was graded according to the National Cancer Institute's Common Terminology Criteria for Adverse Events. There was no treatment related to grade 4 or 5 event, and the average event across all the studies was two (Table 1).

**Table 1:** Extraction table RCC: renal cell carcinoma

Name and year of study	Nature of study	Intervention	Overall response (%)	Adverse reaction (highest reported)
Jonasch et al., 2021 [12]	Open-label non-randomized controlled trial	Belzutifan	62	Grade 4 (unrelated to treatment)
Jonasch et al., 2018 [11]	Open-label non-randomized controlled trial	Pazopanib	53	Grade 5
Jonasch et al., 2011 [10]	Open-label non-randomized controlled trial	Sunitinib	33	Grade 3
Roma et al., 2015 [21]	Retrospective	Sunitinib	64.3	Grade 3
Ma et al., 2019 [22]	Retrospective	Sunitinib, sorafenib, axitinib, pazopanib	40. 25. 33. 0. Total response: 34.4	Grade 4. Grade 4. Grade 3. Grace 3
Oudard et al., 2016 [17]	Non-randomized controlled trial	Sunitinib	Not reported among patients with RCC; hence, it was excluded from the meta-analysis	Not reported on RCC

Discussion

We present the first meta-analysis of systemic targeted therapy in the management of VHL-associated RCC, incorporating available literature evaluating the overall response rate and adverse effects of targeted therapy.

Overall Response Rate

The main systemic therapy for RCC in VHL syndrome targets HIF-2α and MTOR-P13K-HIF-α pathways, and our meta-analysis showed that molecules targeting these pathways are viable non-invasive interventions with a variable response rate and toxicity. VHL-associated RCC managed by HIF-2α antagonists showed a significant response when compared to VEGF inhibitors. A phase II prospective pilot trial of sunitinib, a VEGF inhibitor for RCC in VHL disease, showed 33% partial response, 67% stable disease, and 10% progressive response [23]. This finding was similar to the outcome by Oudard et al. in the phase II trial of sunitinib among the National Expert Center for inherited predispositions to renal tumors (PREDIR) network VHL group [17]. Similarly, pazopanib, a multi-targeted RTK inhibitor, demonstrated a 52% overall response rate with a 3% complete response. While pazopanib was associated with much higher response rates, drug resistance soon developed like sunitinib, and intolerable toxicity precluded its further use in RCC in VHL disease patients [24,25]. Belzutifan, a potent HIF-2α antagonist approved in 2022, was evaluated in phase II prospective clinical trials. A significant anti-tumor response in VHL-associated RCC was observed with 49% best responses (95% CI: 35-62) at the 21.8-month follow-up, which increased to 64% at the 39-month follow-up [26,27]. Although there are no direct comparisons between HIF-2α and VEGFR inhibitors, our subgroup analysis evaluating the response rate between these two systemic therapies shows the superior response rate of the HIF-2α antagonist over the VEGFR inhibitors. This better response could be due to its direct effect on HIF-2α, which is directly implicated in VHL-related tumorigenesis.

Adverse Effects

Although the development of VEGFR inhibitors leads to paradigm shifts in the management of sporadic RCC, their significant toxicity in VHL-associated RCC leads to early therapy discontinuation. A large phase III trial of pazopanib vs. sunitinib among sporadic RCC showed a similar discontinuation rate comparable to the outcome of pazopanib in VHL-associated RCC [25,26]. In an asymptomatic VHL-associated tumor where there is no immediate threat to survival, a balance between efficacy and acceptable toxicity becomes the key factor that influences the choice of targeted therapy.

Treatment with sunitinib in patients was discontinued due to adverse effects, with four patients having grade 3-4 elevated liver enzymes [23]. Similarly, pazopanib was discontinued in 23% due to toxicity despite dose reduction in 90% of patients. More importantly, two patients with non-RCC VHL tumors had intracranial bleeding due to increased VEGF-related vascular friability, thus limiting their role in VHL-associated disease [25]. Cardiovascular, hepatic, and metabolic adverse effects were commonly associated with these anti-angiogenic agents [23,25]. On the other hand, belzutifan was reported by Jonasch et al. to have better tolerability, with most patients having grade 1-2 adverse reactions in anemia, headache, fatigue, and dizziness [12]. The increased risk of anemia was expected because of the HIF-2α inhibitory effect on erythropoietin [21,28,29]. Although 27% had intervention of blood transfusion or growth factors, this did not result in the discontinuation of treatment [22,26]. The most severe adverse event reported was grade 4 in a patient who experienced retinal detachment, along with one death that was considered unrelated to the treatment. Early cessation of treatment was, however, lower in HIF-2α-treated patients compared to anti-angiogenic patients, which could be due to the better tolerability of HIF-2α.

Limitations of This Review

Limited primary data: There is a paucity of controlled studies in the literature with no available randomized studies comparing the outcomes between the different systemic therapies. Most of the included studies were terminated because of poor accrual rates due to the rarity of the disease and the toxicity trigger level. Over-reliance on the open-label studies in our meta-analysis could have influenced our response rate. While there are a few case studies, this was not included as patient eligibility criteria differ, including the mode of diagnosis of VHL in their studies.

Heterogeneity of response definitions: Accessing the effectiveness of any systemic therapy in VHL disease is challenged by the definition of the precise assessment of the growth of lesions and the frequency of intervention required. Equally, there was variability in the eligibility criteria for the studies. Owing to this variability in pre-defined data, we pooled data from studies with inherent factors that potentially can influence their response, and we recognized that this limits the validity of this review.

Publication bias: We included three open-label trials and two retrospective studies, which were individually assessed for publication bias. Visually, the Egger test for asymmetry was not significant. Although there was no evidence of publication bias, exclusion is not possible given the small number of studies.

Implication for Clinical Practice

Despite the above limitations as above, the result suggests that systemic therapy has a role in the management of RCC in VHL disease and patients had better response to HIF-2α.

Implication for Research

With advances in molecular knowledge regarding the genetic alterations in VHL disease, targeted therapy is an alternative to surgery in those with reduced kidney capacity. Prophylactic targeted gene therapy may play a role in preventing the development of tumors associated with this group of patients if genetic alteration is detected.

## Conclusions

This meta-analysis was conducted to assess the overall response rate and toxicity profile of targeted therapeutic agents in the treatment of RCC associated with VHL disease. The results indicate that targeted therapies achieve a clinically relevant overall response rate of 42%, with HIF-2α inhibitors demonstrating superior efficacy and tolerability compared to agents targeting VEGFR. Notably, belzutifan exhibited enhanced therapeutic responses and a reduced incidence of severe adverse events, suggesting its potential to deliver effective tumor control while mitigating renal impairment commonly associated with repeated surgical interventions.

These findings are consistent with the study's aim to evaluate the clinical utility of molecularly targeted treatments in this rare hereditary malignancy. By addressing the pathogenic VHL-HIF-VEGF signaling axis, targeted therapies present a rational and less invasive alternative to surgery, offering the preservation of renal function without compromising oncological outcomes. Nevertheless, the paucity of available studies and the absence of randomized controlled trials underscore the necessity for further rigorous investigations. Future research should prioritize the evaluation of long-term outcomes, optimal sequencing of therapeutic agents, and combination regimens to enhance systemic treatment strategies for VHL-associated RCC.
